# Urolithiasis analysis in a multiethnic population at a tertiary hospital in Nairobi, Kenya

**DOI:** 10.1186/s13104-017-2474-3

**Published:** 2017-04-20

**Authors:** Francis K. Wathigo, Alfred Hayombe, Daniel Maina

**Affiliations:** Aga Khan University Hospital, Nairobi, P.O BOX 30270-00100, Nairobi, Kenya

## Abstract

**Background:**

Urolithiasis is a global problem whose incidence is reported to be on the rise across the world. Previously, urolithiasis was reported as being rare among the indigenous African population but recent data suggest otherwise. This study reviewed the demographic and clinical characteristics of patients with urolithiasis seen at the Aga Khan University hospital Nairobi (AKUHN) as well as the chemical composition of the stones and the modalities of therapy used.

**Methods:**

This was a retrospective study which utilized patients’ clinical and laboratory records from 2013 to 2014. Sixty-seven symptomatic patients with confirmed urolithiasis formed the study. This study aimed to describe the clinical characteristics of patients, modalities of treatment as well as the chemical composition of renal stones from patients diagnosed and managed for urolithiasis during a duration spanning 17 months. Wet chemistry was utilized for analyzing the chemical composition of the urinary calculi. Data on age, sex, symptoms, radiological investigations done, location of the calculi, chemical composition of calculi and therapeutic procedures instituted were extracted and analyzed.

**Results:**

Ages ranged from 3 to 87 years with a median of 42; males were the majority (79%) and the commonest presenting symptoms were flank pain (91%) and dysuria (19%). The majority of the stones were located in the ureters (46%) and at the pelvi-ureteric junction (25%). A statistically significant difference in frequency of lodgment at the pelvi-ureteric site between males and females was noted. However, the number of female patients in this study was small and studies with larger numbers of female participants are required to confirm this observation. All stones contained calcium and oxalate, often as the only constituents (72%). In the remainder of the stones, other constituents such bicarbonate, ammonium, phosphorous, magnesium, uric acid and cystine occurred in varying combinations with calcium oxalate. Laser lithotripsy was the most performed therapeutic procedure (77.6%).

**Conclusions:**

Males formed the majority of patient with urolithiasis. Overall, most of the calculi were located in the ureters except in women where the pelviureteric location was commoner. Stones containing calcium oxalate only were predominant across the age groups and in both sexes. Lithotripsy was the commonest mode of management.

## Background

Urolithiasis refers to a condition characterized by the formation or occurrence of calculi in the urinary tract. The incidence and prevalence rates for urolithiasis vary across different regions of the world with higher rates reported in countries such as Spain and Turkey [1]. A rise in the incidence of urolithiasis has also been documented in United States of America [2]. Urolithiasis in tropical Africa, though less common than in Western world, is increasingly being appreciated as a problem of growing importance. Studies conducted in Kenya and in the larger East Africa region demonstrate arise in the reported cases of urolithiasis in the past 30 years [3, 4]. Underdiagnosis however remains a major challenge [5]. In addition, most of the local studies do not describe the chemical composition of the renal calculi.

Certain factors have been noted to predispose to development of urinary calculi. Metabolic conditions such as hypercalciuria, hyperoxaluria, hyperuricosuria, cystinuria and hypocitraturia have been identified as important risk factors [6, 7]. Age is another risk factor, with a significant rise in incidence of urolithiasis noted after the age of 40 years [1, 8]. Gender is yet another significant risk factor with men predisposed to developing urolithiasis compared to women [1, 8]. Race has also been proposed to be significant with higher rates noted in Caucasians compared to African-Americans and Asians [1, 9]. Aberrations in urinary pH, as well as presence of urinary tract infections are additional factors that have been implicated in urolithiasis.

Various studies investigating the composition of renal stones in African populations revealed calcium oxalate as the commonest compound across the various age groups [10–12]. The prevalence of calcium oxalate calculi in adults has been shown to be comparable between industrialized and non-industrialized countries but purine and struvite stones are commoner in the non-industrialized countries [13].

In previous studies conducted in Kenya, the commonest presenting clinical features in patients with urolithiasis were pain and haematuria while the commonest modes of imaging were ultrasonography and plain abdominal radiographs [4]. The majority of calculi were located in the renal pelvis and ureters with extracorporeal shock wave lithotripsy (ECSWL) the commonest mode of treatment [3].

Various methods are available for stone analysis and they include wet chemical analysis, thermogravimetry, scanning electron microscopy, optic polarizing microscopy, spectroscopy, infrared spectroscopy, X-ray powder diffraction and elementary distribution analysis. Wet chemical analysis is still the most widely utilized technique for calculi analysis in clinical routine laboratories in Africa [14]. This method has lower costs of operation but is time-consuming and only suitable for relatively large stone specimen. In addition, it has the disadvantage of only being able to identify the presence of individual ions without differentiating specific compounds. [14].

This study aims to describe the clinical characteristics of patients, modalities of treatment as well as the chemical composition of renal stones from patients diagnosed and managed for urolithiasis during a duration spanning slightly over 1 year, utilizing wet chemical analysis.

## Methods

This was a retrospective study conducted at AKUHN, a teaching hospital serving a multi-ethnic population, in which patients’ clinical and laboratory records were reviewed. Clinical and laboratory records for all patients with confirmed urolithiasis at AKUHN, during the period spanning from January 2013 to May 2014 were included: sixty-seven symptomatic patients with confirmed urolithiasis formed the study. Data relating to demographic characteristics, clinical features, modalities of diagnosis and treatment were extracted from the clinical records. Data concerning stone composition was extracted from corresponding laboratory records.

The analytical method used to analyze stones at AKUHN was wet chemistry. In brief, the stones were pulverized into fine powder and mixed with different liquid reagents to detect various chemical components through observation of effervescence and color changes.

In statistical analysis, continuous variables were expressed as means and medians. Categorical data were summarized into percentages. Differences in categorical variables between groups were assessed using Chi square test or Fisher’s exact test as appropriate. P values less or equal to 0.05 were interpreted as statistically significant. Statistical analysis was performed using SPSS Statistics software version 22 (IBM, Armonk, USA).

## Results

The ages ranged from 3 to 87 years with a median of 42 years and a mean of 43.5 years (SD 17). Only 3 patients (4.5%) were aged below 18 years. Males were the majority comprising 79% of the patients. The commonest clinical features were flank pain (91%), dysuria (19%), nausea/vomiting (15%) and haematuria (15%). The majority of the patients (92.5%) had only one site of lodgment involved. The ureters and the pelvi-ureteric junction were the anatomical sites most commonly affected. The bladder and urethra (11.3%) were less commonly involved (Table 1).

**Table 1:** Demographic and clinical characteristics of patients

Demographic and clinical characteristics	
Characteristic	(n = 67)
Age, median (years)	42
Male, n (%)	53 (79.1)
Female, n (%)	14 (20.9)
Presenting clinical features, %	
Flank pain	91
Dysuria	19
Nausea/vomiting	15
Microscopic haematuria	15
Fever/chills	10.4
Anatomical sites involved in lodgment of calculi, %	
One site	92.5
Two sites	7.5
Anatomical site of lodgment of calculi, %^a^	
Ureter	46.5
Pelvi-ureteric junction	25.4
Vesicoureteric junction	16.9
Others	11.3
Imaging modalities, %	
Computerized tomography (CT KUB)	81
Abdominal/pelvic ultrasonography	15
Micturating cystourethrogram	4

^a^n = 71 because four patients had calculi located in two different anatomical sites

Among the imaging studies performed, computerized tomography of kidney, ureters and bladder (CT KUB) was the most commonly used mode of evaluation (Table 1).

With regards to chemical composition, calcium and oxalate were present in all the stones. The majority of the calculi (71.6%) contained only calcium and oxalate. Stones containing only bicarbonate and calcium oxalate as a combination constituted 22.4% of all calculi. Calculi containing other constituents such as ammonium and cystine were uncommon with combined total of less than 10% (Table 2).

**Table 2:** Composition of urinary calculi

Constituents, n (%)	(n = 67)^a^
Calcium oxalate only	48 (71.6)
Calcium oxalate + bicarbonate	15 (22.4)
Calcium oxalate + ammonium	1 (1.5)
Calcium oxalate + phosphate	1 (1.5)
Calcium oxalate + ammonium + bicarbonate	1 (1.5)
Calcium Oxalate + ammonium + bicarbonate + phosphate uric acid + cystine	1 (1.5)

^a^Multiple specimens submitted to the laboratory for a single patient from one surgical procedure were processed as one sample

Stones containing only calcium oxalate predominated in both genders as well as in both age groups (Table 3).

**Table 3:** Composition of calculi by age and gender

Composition	Age^a^			Gender		
n (%)	<42 years (n = 32)	≥42 years (n = 35)	*P* value	Male (n = 53)	Female (n = 14)	*P* value
Calcium oxalate only	22 (68.8)	26 (74.3)	0.51	40 (75.5)	8 (57.1)	0.74
Calcium oxalate + bicarbonate	9 (28.1)	6 (17.1)	0.23	12 (22.6)	3 (21.4)	0.50
Others	1 (3.1)	3 (8.6)	0.62	1 (1.9) (1)	3 (21.4)	1.00

^a^The patients were divided into two groups based on the median age

There was no statistically significant difference in composition of calculi by gender or by age (*P* > 0.05, Table 3).

The ureters were the commonest sites of lodgment in both age groups as well as in males (Table 4). There was no significant statistical difference noted in the location of calculi between the two age groups (*P* > 0.05). However, in females, the pelviureteric junction was the commonest site of lodgment (64.3%) and this was statistically different when compared to males (*P* = 0.01).

**Table 4:** Location of calculi by age and gender

Location	Age^a^			Gender		
n (%)	<42 years (n = 33)^b^	≥42 years (n = 38)^b^	*P* value	Male (n = 57)^b^	Female (n = 14)^b^	*P* value
Ureter	16 (48.5)	17 (44.7)	0.72	28 (49.1)	5 (35.7)	0.26
Pelvi-ureteric junction	7 (21.2)	11 (28.9)	0.46	9 (15.8)	9 (64.3)	0.01
Vesicoureteric junction	5 (15.2)	7 (18.4)	0.72	12 (21.1)	0 (0)	0.58
Bladder	3 (9.1)	3 (7.9)	0.85	6 (10.5)	0 (0)	0.33
Urethra	2 (6.1)	0 (0)	0.21	2 (3.5)	0 (0)	0.26

^a^The patients were divided into two age groups based on the median age

^b^Total n = 71 because four patients had calculi located in two different anatomical sites

The majority of the patients with urolithiasis (77.6%) were managed with laser lithotripsy. Among those patients who underwent lithotripsy, a minority (13.5%) also had Dormia basket extraction done concurrently with lithotripsy. Dormia basket extraction (without concurrent laser lithotripsy) was carried out in 7.5% of the patients while PCNL was undertaken in 9% of the patients. In 38.8% of the patients, stenting was also undertaken in addition to the other surgical procedures required to address the urinary calculi (Table 5). All the patients had positive outcomes and were subsequently discharged from hospital following treatment.

**Table 5:** Modes of treatment for urolithiasis

Modes of treatment for urolithiasis	
Frequency of modality used, n (%)	(n = 67)
Ureteroscopy/cystoscopy and laser lithotripsy	52 (77.6)
Ureteroscopy/cystoscopy and Dormia basket extraction	5 (7.5)
Percutaneous nephrolithotomy (PCNL)	6 (9)
Cystolithopaxy	1 (1.5)
Open nephrostomy	1 (1.5)
Spontaneous passage of calculi in urine	3 (3)
Patients in whom stenting was also done	26 (38.8)

## Discussion

Our study demonstrated that the majority of patients with urolithiasis were male and that the commonest presenting feature was flank pain. These findings are consistent with previous studies from various parts of the world [1, 3, 4]. It therefore seems advisable that male patients presenting with flank pain be properly evaluated for urolithiasis, as various studies have alluded to underdiagnosis being of major concern [5]. The majority of the stones were located in the ureter and pelviureteric junction. Again, these findings are consistent with a previous study by Ngugi et al. that showed the ureter and renal pelvis to be the commonest anatomical sites of involvement [3]. There was no statistically significant difference in anatomical location of lodgment between the two age groups analyzed. A statistically significant difference in frequency of lodgment at the pelviureteric site between males and females was noted (*P* = 0.01). However, the number of female patients was quite low [14]. Studies with larger numbers female participants are required to confirm this observation.

The major constituents of the stones were calcium and oxalate, findings which are in keeping with previous studies conducted across various parts of the world [7, 10, 11]. There was no statistical difference in chemical composition of the stones when comparing patients by age or gender.

This study goes beyond previous local studies by including analysis of the chemical composition of the renal calculi. Information on the composition of renal calculi is important in understanding the pathophysiology of urolithiasis. For instance, struvite stones usually occur against a background of urinary tract infection while uric acid stones tend to form in unduly acidic urine [7]. Information on the chemical composition of stones may also influence mode of therapy chosen: brushite (calcium hydrogen phosphate dihydrate) and cystine stones are harder and therefore more resistance to shock wave lithotripsy [15, 16]. Similarly, chemical agents such as sodium citrate or potassium citrate may be used to alkalinize urine as part of medical management in patients with uric acid stones [16, 17].

Laser lithotripsy was the commonest modality of treatment which again is consistent with the results of the study done by Ngugi et al. [3].

One drawback of this study was the method of stone analysis utilized. Wet chemical analysis suffers the handicap of only being able to identify the presence of individual ions without differentiating specific compounds in different stone types and mixtures. As a result, this method will not, for example differentiate between calcium oxalate monohydrate and calcium oxalate dihydrate stones. Another limitation is the relatively small sample size involved in this study.

## Conclusions

We analyzed renal calculi from sixty seven patients using wet chemistry technique. Male patients were the majority with a male to female ratio of 3.8:1. Overall, the majority of the calculi were located in the ureters except in women where the pelvi-ureteric location was the commonest. A statistically significant difference in frequency of lodgment at the pelvi-ureteric site between males and females was noted. Stones containing calcium oxalate only were predominant across the age groups and in both sexes. Larger studies are however recommended to confirm this observation due to the small number of female patients involved in this study. Lithotripsy was the commonest mode of management.

## Abbreviations

AKUHN: Aga Khan University Hospital Nairobi
CT KUB: computerized tomography of kidney, ureters and bladder
ECSWL: extracorporeal shock wave lithotripsy
PCNL: percutaneous nephrolithotomy
SD: standard deviation
